# The impact of the COVID-19 pandemic on global neurosurgical education: a systematic review

**DOI:** 10.1007/s10143-021-01664-5

**Published:** 2021-10-08

**Authors:** Raunak Jain, Raquel Alencastro Veiga Domingues Carneiro, Anca-Mihaela Vasilica, Wen Li Chia, Abner Lucas Balduino de Souza, Jack Wellington, Niraj S. Kumar

**Affiliations:** 1 Department of Medicine, UCL Medical School, 74 Huntley St, London, WC1E 6DE UK; 2 Universidade Federal de Santa Catarina, Florianópolis, Brazil; 3 Cardiff University School of Medicine, Cardiff, UK; 4 Universidade Evangélica de Goiás, Anápolis, Brazil

**Keywords:** COVID-19, Neurosurgery, Education, Pandemic, Global surgery

## Abstract

The COVID-19 pandemic has disrupted neurosurgical training worldwide, with the shutdown of academic institutions and the reduction of elective surgical procedures. This impact has disproportionately affected LMICs (lower- and/or middle-income countries), already burdened by a lack of neurosurgical resources. Thus, a systematic review was conducted to examine these challenges and innovations developed to adapt effective teaching and learning for medical students and neurosurgical trainees. A systematic review was conducted in accordance with the Preferred Reporting Items for Systematic Reviews and Meta-Analysis Protocols (PRISMA-P) and The Cochrane Handbook of Systematic Reviews of Interventions. MEDLINE, PubMed, Embase and Cochrane databases were accessed, searching and screening literature from December 2019 to 5th December 2020 with set inclusion and exclusion criteria. Screening identified 1254 articles of which 26 were included, providing data from 96 countries. Twenty-three studies reported transition to online learning, with 8 studies also mentioned redeployment into COVID wards with 2 studies mentioning missed surgical exposure as a consequence. Of 7 studies conducted in LMICs, 3 reported residents suffering financial insecurities from reduced surgical caseload and recession. Significant global disruption in neurosurgical teaching and training has arisen from the COVID-19 pandemic. Decreased surgical exposure has negatively impacted educational provision. However, advancements in virtual technology have allowed for more affordable, accessible training especially in LMICs. Using this, initiatives to reduce physical and mental stress experienced by trainees should be paramount.

**Supplementary Information:**

The online version contains supplementary material available at 10.1007/s10143-021-01664-5.

## Introduction

Neurosurgical care is unequal worldwide, with up to five billion people from low- and middle-income countries (LMICs) not having access to safe and affordable neurosurgery in a year [1]. The lack of neurosurgeons in LMICs is one of the main issues, whereby a maldistribution of neurosurgeons in different continents and countries results in a disproportionate number of patients treated by far fewer neurosurgeons in LMICs compared to high-income countries (HICs) [2]. Therefore, the training of neurosurgeons is of utmost importance to transform the landscape [2] and reduce the aforementioned burden. Multiple approaches, such as visiting fellowships to HICs, have been taken to mitigate this issue [3].

However, the novel coronavirus (COVID-19) pandemic has imposed several restrictions to daily life along with changes in healthcare, challenging previous models of neurosurgical training and bringing forth the necessity to adopt new technologies in response [3]. Literature has indicated changes in neurosurgical practice: with reduced influx of procedures and restrictions to emergency and essential interventions [4, 5], and reductions in available surgical training opportunities, as well as impacts on continued education for both trainees and medical students [6]. In face of this, technologies such as teleconferences, online learning, telemedicine, simulations [6] and immersive technology [3] have been pointed out as possible solutions for learning and training during the pandemic.

Nonetheless, the previous disparities between LMICs and HICs may have been further enhanced. Therefore, the impacts and disparities, in neurosurgical education and training, need to be properly addressed and understood in the pandemic context, in order to provide solutions. But, to the best of our knowledge, literature analysing neurosurgical training impacts, both to LMICs and HICs, and the solutions provided in these countries is scarce.

Thus, we conducted a systematic review in order to assess the impacts of the COVID-19 pandemic on neurosurgical training to both LMICs and HICs. We also aimed to discern the alternatives implemented within these countries and challenges faced in this context. This knowledge will be helpful to comprehend the possible solutions and prevent an even larger burden to neurosurgical access due to impaired training, both in face of the ongoing COVID-19 pandemic in LMICs as new coronavirus variants evolve, especially within India and Brazil, and in regard to similar future events.

## Materials and methods

This systematic review has been conducted in accordance with the Preferred Reporting Items for Systematic Reviews and Meta-Analysis Protocols (PRISMA-P 2015) [7].

### Eligibility criteria

Article selection was conducted according to the following inclusion criteria: (i) studies exploring the impact of the COVID-19 pandemic on neurosurgical training provision for residents or medical students; (ii) studies exploring neurosurgery curriculum adaptations; (iii) studies exploring changes in training programmes structure; (iv) studies published between December 2019 and 2020; and (v) studies published in peer-reviewed journals, in English language.

Letters to the editor, editorials, commentaries, opinion pieces and conference abstracts have been excluded.

### Search strategy and information sources

The following databases have been searched for articles: Medline, PubMed, EMBASE and Cochrane. The review period spanned November 15th, 2020, and December 5th, 2020. The full search strategy can be found in the supplementary material (supplement 1).

### Study records and data management

Literature search results were imported into Mendeley reference manager (Version 1.19.4; Elsevier, 2019) for article selection and deduplication.

### Selection process

Titles and abstracts of retrieved articles were independently screened by two authors for each database, based on eligibility criteria. The results were pooled and the full text for all potentially relevant publications were retrieved and reviewed. Any disagreement with regard to article eligibility was resolved with consensus.

### Data items

Extracted relevant data were compiled in a Microsoft Excel spreadsheet (Microsoft Excel, Microsoft Corporation, 2010) and data items included were as follows: author(s); year of publication; title; country; subspecialty of neurosurgery (if applicable); methods of study; difficulties caused by the pandemic, type of/level of neurosurgical education which was affected (medical students/neurosurgical trainees or residents), methods used to cope with these difficulties; attitudes of medical students and neurosurgical trainees; solutions implemented to provide neurosurgical teaching and training; and any other information deemed relevant.

### Outcomes and prioritisation

The main outcome of this systematic review was to identify the difficulties that have been provoked by the COVID-19 pandemic in relation to neurosurgical education and practical theatre experience for trainees and medical students across the world. The secondary outcomes were threefold: (i) to explore how online/telecommunication methods have been used to supplement education during the pandemic; (ii) to examine the differences and describe the use of telecommunication/online/other innovative methods in teaching in HICs and LMICs; and (iii) to evaluate and suggest solutions for the future and explore the possible reasons for discrepancies between HICs and LMICs.

### Risk of bias in individual studies

The included articles were assessed using the Oxford Center for Evidence Based Medicine (OCEBM; version 2.1) for level of evidence.

### Risk of bias across studies

A meta-analysis has not been undertaken with regard to the heterogeneity present in the methodologies and study designs present over the above articles.

## Results

**Fig. 1:**
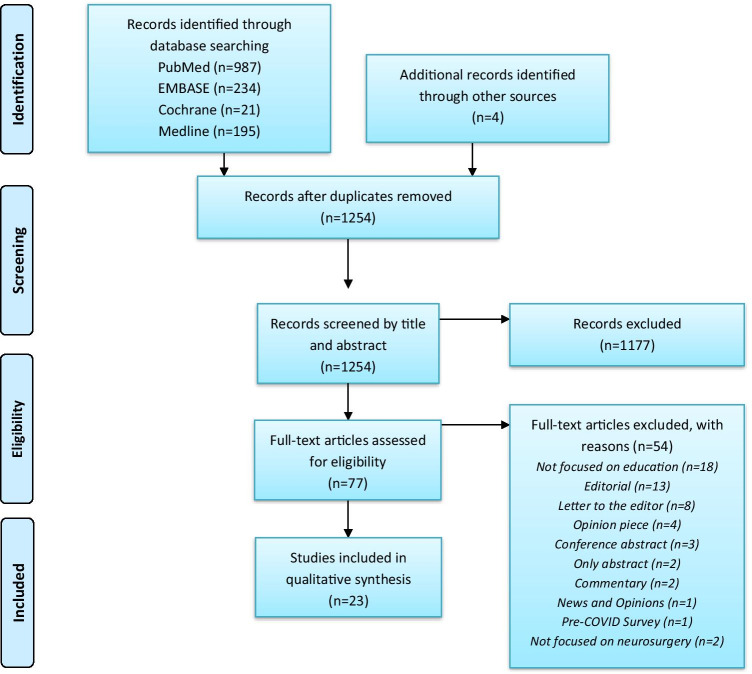
PRISMA flow diagram depicting selection and screening process

Following deduplication, 1254 articles were screened. After the titles and abstracts were examined, 1177 articles were excluded as they did not focus on the impact of COVID-19 on neurosurgical teaching or training. A further 19 were excluded because they were not focused on neurosurgical education, and another 34 excluded as they comprised weak evidence—e.g. editorials, letters to the editor. Overall, this systematic review collated data and information from 26 articles, with 15 being based on surveys (Fig. 1). The collated data of impacts to education and solutions have been summarised in brief with a table in the supplementary material (Supplement 2).

### General impacts on neurosurgical trainees’ education

In almost all neurosurgery departments, teaching, academic meetings, lectures and conferences were converted to an online format during the course of the pandemic. An emphasis was laid upon reducing inter-individual contact at the expense of exposure to the operating room [8–12]. However, reducing such real-world experience has had detrimental effects on resident training.

#### Practical skills training

Surveys of residents from both HMICs and LMICs reported decreased operating volumes, resident participation in operative procedures and time spent in the OR [4, 10–17]. A survey done with trainees in 5 Southeast Asian nations showed a significantly greater reduction in elective cases in Indonesia and the Philippines (*P* < 0.001), the latter of which also contained the most respondents (76%), reporting closure of outpatient clinics, with the median duration of closure being 8 weeks [18]. This reduction was shown to negatively impact neurosurgical trainees, especially regarding the reduced quantity and variety of cases available. This led to reduced procedural skills’ subspecialisation, reduced responsibilities, poor independent management and hospital access [15, 19–22]. In some centres, these reductions in case load have been more apparent for residents, with one US teaching hospital observing a 59% decrease in bedside procedures for residents when compared with previous years [16]. However, in other neurosurgical departments, the number of elective procedures has decreased irrespective of the stage of training [18]. Concerning this decrease, there is a disparity between the opinions LMICs and HICs trainees hold regarding how this reduced operative volume may impact their training. Over 62% of neurosurgical residents in a US survey did not believe that the decrease in operative volume would negatively affect their surgical abilities [10]. This is contrasted by survey evidence in LMICs within countries such as Indonesia and the Philippines where over 75% of neurosurgical trainees believe that the COVID-19 crisis will have a negative effect on their training, primarily due to the lack of hands-on experience [18].

Additionally, due to the COVID-19 pandemic, the shortage of medical professionals led to many being reassigned from specialised neurosurgical wards into ‘COVID wards’. Residents were more likely to be redeployed to general COVID wards in LMICs, for example in southeast Asia where more than 36% of the neurosurgical trainees were deployed to COVID-19 units according to a multiple-country survey of Indonesia, Malaysia, the Philippines, Singapore and Thailand [18]. More than 60% of these trainees reported concerns on the lack of training in managing COVID-19-positive patients [18]. Alongside this, the pandemic also translated into at least one missed opportunity per individual resident for international training such as elective rotations or observership programmes, conference presentations and clinical fellowship positions [18].

A positive aspect, however, emerged with the aforementioned reduction in elective cases. The cancellation of elective procedures, which in one case was compensated with a greater focus on remote research. In the USA, 90% of surveyed participants reported that COVID-19 resulted in increased time dedicated to research and publishing, resulting in continued learning and development and contributing to the pool of scientific research that is available [10]. Other reports in the country showed that all the junior residents had declared that they would work remotely on research projects [12]. In Italy, for example, the reduced time spent in the neurosurgical department has been effectively employed for the purposes of studying, scientific work being finalised and projects being completed and submitted for publications. In 55.7% of residents, this has translated into an increase in research output, despite the pandemic [23]. However, in LMICs, academically oriented residents were more likely to be transferred to general COVID wards, rather than engaging in research, with 33% of residents missing out on publications and conference presentations [18].

#### Shift to online teaching

In-person teaching activities were cancelled in most neurosurgical departments worldwide [8–12, 24]. There has been a greater use of online education initiatives, including webinars, virtual conferences and lecture series [8]. In addition, there has been a rapid increase in the popularity of telemedicine and virtual teaching platforms, such as Zoom and Microsoft Teams [25]. The use of weekly conferences held virtually on zoom, with live presentations, has further improved learning and education [16]. Online streaming of procedures was also initiated in some training programmes [14]. Other programmes also took advantage of surgical videos and encouraged attendees to create their own mock oral board cases. Weekly ward round and grand round discussions were kept and in one case made virtual [15]. To allow for interaction between residents and fellows in the era of social distancing, virtual meetings were organised to discuss new initiatives, cases and medical practice [15]. For many neurosurgical trainees, this technological shift improved access to educational resources. Some trainees participated multiple times in virtual conferences multiple times each week, suggesting that virtual education could partially substitute in-person sessions [26]. However, several cross-sectional studies, done to assess this shift in education, illustrated a pattern [11, 13, 15]. In North America, which is considered to have better socioeconomic status, 98.5% of respondents said that their programme was transferred to online platforms [15]. In stark contrast, on the African continent, only 43.9% of respondents reported a shift to online teaching and 12.2% to online national teaching [11]. Similarly, in India, a major shift to online teaching occurred, reported by 61.0% of respondents [13]. Thus, in comparison, LMICs lagged behind HICs in the provision of online education and new methods of teaching, emphasising the global inequality in neurosurgical education [15].

The positive side in this shift was that trainees and medical students alike have responded positively to the introduction of virtual conferences. For example, 92.0% of participants that attended the Virtual Global Spine Conference (VGSC) deemed the content as valuable, and 94.0% would continue attending these conferences post-COVID [25]. In one instance, at a tertiary centre in India, 84.6% of attendees felt that virtual learning and webinars had increased their participation [27]. Residents in developing countries usually have great difficulties in attending foreign neurosurgical conferences, workshops and teaching due to poor funding. Hence, virtual methods of education have provided residents with a cost-effective, accessible means to attend such learning events [4].

Attendance for virtual webinars has also grown significantly. A survey across multiple centres in Saudi Arabia demonstrated that the attendance for teaching has increased from 78.2% during in-person teaching to 89.1% using online webinars [8]. However, the online learning experience differed across institutions in a study from India, with academic sessions lowered by 60% (from 5 to 2 weekly), research work being affected and conferences being cancelled in training centres [13]. In the African continent, a survey of neurosurgical residents reported that 57.7% of respondents experienced postponed exams, and 19.5% experienced cancelled exams [11]. Across the USA, a survey of neurosurgical residents from 8 residency programmes found that 90.0% of programmes were solely delivering online teaching in the first months of the pandemic [10]. Similar findings from a separate survey of residents from 29 US states found that of the programmes that shifted teaching to electronic platforms (98.5%), 71.9% made this change within 1 week of restrictions taking effect [15]. However, findings from a survey of residents in India revealed that 13.6% of respondents reported the cessation of academic sessions without in-person or videoconference alternatives at the time of survey [13]. Despite 75.3% of surveyed neurosurgical trainees in Saudi Arabia agreeing that online webinars were more useful than traditional lectures, only 39.6% felt that webinars should replace face-to-face teaching [8].

Additionally, the virtual education adaptations implemented in training programmes were ineffective in teaching residents the manual dexterity demanded in the operating theatre, as well as the pre- and post-operative management of patients in a clinical setting [22, 27].

### Neurosurgery residency programme adaptations and solutions

In face of the pandemic and the changes to neurosurgical practice, several adaptations have been made in neurosurgical residency programmes. A neurosurgery department from Pennsylvania reported a reduction in neurosurgical resident groups and operating room participants by half, with only one resident allowed per day. Additionally, to control the spread of the pandemic and promote surgeon well-being, neurosurgeons were alternating weeks on and off, and were restricted to certain types of surgery, for example, not being allowed to participate in procedures requiring sinus drilling [14]. In addition, a US academic tertiary centre has reported that services were restructured to minimise the number of residents at any given time, with a reduction in rotations and a downsizing of daily rounds and covering teams by half [19].

Similar changes have been reported in other countries. In Italy, a survey targeted at neurosurgical residents showed that no residency programmes were stopped but 63.5% of respondents faced a reduction in work time imposed by educational providers. Outside neurosurgical departments, residents were reported to have helped the national health system in the management of COVID-19 patients when needed [23]. Egyptian researchers have also conducted a national survey which indicated that 10% of respondents were deployed to other departments involved in the management of COVID-19 patients. Fifty-six percent also reported a reduction in the number of residents scrubbed in for the same surgical procedure [4]. In a survey evaluating neurosurgical training in the African continent, 25.2% of respondents had their training rotations suspended [11].

In order to maintain neurosurgical education and training, there was a shift to online-based education. In the USA, webinar-type online platforms such as Zoom have been utilised for lectures and conferences [16, 25], mentoring sessions [28] and surgery live-streaming [29]. In the operating theatre, camera-embedded surgical lights were routed to an in-room audio-visual interface to keep residents in contact with the operating room [14, 29]. To maintain surgical hand skills education at a distance, an at home microsurgical skill training programme was developed and implemented in Argentina, in which participants used a set of instruments and smartphones as magnification devices in order to perform several exercises—explained in advance via video [20]. In addition, in the USA, a 3D printed low-cost and open-access simulation device of the lumbar spine was developed for anatomical learning and spine surgical simulation, allowing neurosurgical trainees to learn remotely [30].

### Impacts on medical students’ neurosurgical education

COVID-19 has had a drastic impact on medical student education in neurosurgery—rotations and elective courses have been cancelled; there has been a lack of in-person exposure to operating theatres and in-person seminars and conferences have been suspended [8]. Hence, there has been a push towards making these teaching resources available online [8].

Following virtual education, students had improved anatomical understanding and enjoyed the real-world interaction with the surgical team. This enabled active learning, thus better educational outcomes [29]. Virtual conferences through video-streaming platforms have improved medical student access to educational content and students from different countries could attend free of charge—requiring only an internet connection [28]. However, virtual reality was trialled as part of a neurosurgical elective in the Case Western Reserve University [5].

### Medical students and neurosurgical trainees perspective and mental health

#### Medical students

Whilst neurosurgical rotations were cancelled in some departments, in university hospitals, there have been new initiatives to improve medical student learning, enthusiasm and experience [29]. This included the aforementioned live streaming of surgical procedures via Zoom but also a training camp aimed at medical students [28]. This covered aspects ranging from mentorship, to networking and interview preparation tips [28]. The enthusiasm related to the event was reflected in the positive feedback received: 100% of students desired that the event run annually, as it decreased the concerns associated with sub-internships and the interview process (79.8% of participants) [28]. It also developed neurosurgical knowledge in 65.0% of students [28]. As to a neurosurgical elective that incorporated virtual reality, 92.0% of medical students strongly agreed that this was a valuable learning experience and that it improved anatomical understanding, with all recommending it [5].

#### Neurosurgical trainees

The restrictions imposed by the pandemic on in-person teaching and working in hospitals have resulted in some unexpected benefits. There has been a reported increase in educational and scientific activities undertaken by neurosurgical trainees outside of the hospital. Furthermore, time outside clinical duties has been invested into watching lectures remotely, conducting research and preparing for future examinations [15, 23].

The negative implications of the pandemic have become apparent among trainees. Many have expressed a fear of their limited competencies when compared to colleagues with pre-COVID training [13]. The effects of the restrictions imposed by the pandemic have been observed not only in the professional life of neurosurgical residents, some of whom have experienced burnout syndrome, but also in their personal life and relationships, which have been negatively affected [4].

Trainees in the USA, although not reportedly concerned about the completion of their fellowships, manifested a slight concern with regard to their future jobs [17]. Conversely, the general concerns of neurosurgical trainees in Asian LMICs have been revolving around ideas related to career progression, examinations being deferred and to decreased training in terms of the clinical and operative skills required in the operating theatre [13, 18]. Another study assessed the impact of virtual learning (VL) for trainees in India [27]. It concluded that VL, whilst improving participation and communication with other health care professionals, cannot entirely cover the practical component required in a surgical profession—with only 15.4% of survey respondents acknowledging an improvement in their skills following these sessions [27].

A survey of current fellows found that 95.5% were not worried about the impact of COVID-19 on the successful completion of their fellowships [17]. However, 25% were concerned about the impact of COVID-19 towards the start of their future jobs [17]. A total of 13.6% of surveyed fellows did not have a job lined up after fellowship, and 22.7% reported job interview cancellations due to COVID-19 [17]. Similarly, another survey of residents from 29 US states showed that 26.5% of respondents expressed concerns over COVID-19 limiting their ability to obtain employment and/or fellowships of their choice in the future [15].

In terms of the cumulative residency experience, senior residents in the USA were more likely than their junior colleagues to report the negative influence of COVID-19 on their residency experience (43.8 vs 26.7%, *p* = 0.028) [15]. Similar sentiments were found in a survey of 5 neurosurgical tertiary centres in Egypt as 91.3% of senior residents thought residency training should be extended, in contrast to 18.5% of junior residents surveyed [4].

Mental health was affected during this time with reports that 90% of trainees assessed in a survey comprising both LMICs and HICs had mental health impacts due to COVID-19 [9]. In Egypt, 68% of residents experienced burnout symptoms in the period [4]. Factors such as fear of contracting COVID-19, spreading it to family and friends, uncertainty surrounding the pandemic and reduced social contact were reported as the main stressors [4, 18].

## Discussion

The restrictions imposed by the COVID-19 pandemic have challenged previous models of neurosurgical training, bringing forth the necessity to adopt emerging technological solutions [3]. Our results identified cancellations of elective programmes, reduced real-life theatre experience and suspension of in-person teaching as major concerns. Additionally, trainees have had reduced clinical experience, combined with increased stress and mental health issues. Virtual education and simulation-based training have been pointed out as possible interventions.

Before the pandemic, neurosurgical training was already a significant barrier contributing to the maldistribution of neurosurgeons and care, especially in LMICs [2]. Within the context of the pandemic, traditional training has suffered greatly due to reduced opportunities, which has contributed to the widening of existing inequalities in surgical care. In our systematic review, 10 studies based on surveys of residents, from both LMICs and HICs, reported a decrease in operative volumes, reduced participation in procedures and less time spent in the OR, possibly due to sanitary and virus control actions taken by departments. The deployment of neurosurgical trainees to COVID-19 or non-specialty care was reported in both LMICs and HICs. Dedeilia et al. have also reported similar changes to medical and surgical education in general, describing reduced training opportunities and trainee redeployment to other departments [6]. Furthermore, we found that the modifications made by neurosurgical departments mainly included the following: reduction in the number of rotations, smaller teams, reduction in surgical participation for residents and fewer elective neurosurgical cases. Previous literature has also shown a trend of reorganisation of neurosurgical practice and education [6, 31, 32].

These changes, besides aggravating traditional training opportunities, may have deepened known disparities between LMICs and HICs [3], due to the concentration of resources and technology. In this review, differences between these groups of countries have primarily concerned interventions to continue education and neurosurgical practice. We found that the uptake of online teaching methods has been lower in LMICs, such as India than in HICs such as the USA [10, 13]. Also, a disparity between trainee concerns was found. In the USA, most residents did not believe that the decrease in operative volume would negatively affect their surgical abilities [10], whereas in Indonesia and in the Philippines, the majority of trainees stated otherwise [18]. The disparity in trainee attitudes suggests that trainees in LMICs were less likely to be impressed with the quality or quantity of provision of alternative learning, possibly valuing the on hands practice gained from live surgeries much more than surgeons in HICs.

However, even prior to the COVID-19 pandemic, distance electronic learning tools [33] and immersive technology [3] have been widely pointed out as solutions to significantly decrease the burden of unmet surgical needs worldwide. The Lancet Commission has also recognised that novel technologies are central to the improvement of surgical care in the world [34]. With the COVID-19 pandemic, other solutions such as the visit-based model [33] had to be suspended, bringing forth the necessity to enhance and quickly adopt new technologies. Though cost serves as a frequent barrier in LMICs and disparities were found within this study, many of these interventions are designed to use existing equipment and software, allowing for easy, cost-effective implementation [3, 33]. Simulation-based approaches, intraoperative tele-mentoring and tele-proctoring, so-called immersive technologies, have been largely implemented to aid training in LMICs with the use of existing equipment, such as low-cost mobile devices [3]. Additionally, innovation of online resources has brought new avenues for trainee and student learning and access democratisation. Students who would struggle to afford travelling for conferences abroad or to teaching hospitals can now attend teaching from home cheaply or for free [4]. However, adaptations to each country’s individual neurosurgical training programmes as well as international cooperation are a necessity for quick and effective implementation. For example, collaboration with surgeons from LMICs could widen participation from poorer countries in e-learning programmes [3, 33]. Thus, the pandemic has accelerated the adoption of technological solutions aiding to reduce disparities in training and neurosurgical care and reducing the need for trainee displacement.

In our review, we observed that 77% of studies mentioned online education as a solution to continue training, and in all of these, trainees and medical students have found this method useful. In addition to webinar-type platforms for lectures and seminars, tools such as open anatomical teaching software and surgical live-streaming were implemented to maintain contact with the operating room and aid teaching of surgical techniques. Even before the pandemic, live surgery streaming was used to provide real-time surgical mentoring and interaction between people in different geographic locations and it has been a growing practice in surgery globally [3]. However, this does not compensate for teaching hands-on surgical techniques; thus, other solutions were needed. Some studies point to alternative simulations, such as 3D printed simulation devices and a smartphone centred microsurgical skill training programme, in order to maintain training of manual skills [20, 30]. Simulation has been demonstrated to be highly beneficial to improve procedural knowledge and technical skills, with a growing trend of adopting neurosurgical simulators [35]. The upside is that the so-called immersive technologies, such as virtual simulation, can often be implemented with the use of existing equipment [3]. This makes these technologies more accessible to all levels of training and more adaptable to different countries’ realities. The aforementioned microsurgical skill training programme, for example, was based in Argentina and used the students’ own smartphones as magnification devices [20].

Additionally, in the face of the challenges imposed by the pandemic on neurosurgical trainees, mental health and well-being also need to be properly addressed. In this study, we found that high percentages of trainees have been found to suffer from the impact of COVID-19 on their mental health. In a worldwide survey, 90% of trainees had mental health impacts due to COVID-19, and in Egypt, Ashry et al. reported that 68% of residents experienced burnout symptoms during the pandemic [4, 9]. Factors such as the fear of contracting COVID-19 and spreading it to family and friends, uncertainty surrounding the pandemic and reduced social contact have all been indicated as stressing factors in literature [4, 18]. Indeed, in agreement with our results, other studies have pointed out a decrease in mental well-being for surgical trainees in general and worldwide, with increased stress and burnout rates, negative impacts on interpersonal relationships, home-life disruption and concerns on educational experience [36]. By examining these factors, our results provide data for the implementation of new solutions by neurosurgical departments. Social support for trainees, adequate rest breaks during rounds, reduction and optimization of workload could all aid in the matter.

### Study limitations

The rapid onset and spread of the COVID-19 pandemic resulted in many articles being published to explore its impact on neurosurgical education. Hence, there is a need for systematic reviews to collate and qualify all this evidence together.

Only RCTs, systematic reviews and meta-analyses were considered, thus strengthening the quality of this review. Despite the potential for scarcity of literature whilst screening in this fashion, 26 qualifying articles were found, thus ensuring that there was enough information or data. However, 56% of the included studies involved the use of surveys. Some of these surveys could have been affected by biases, such as voluntary response bias. Additionally, these surveys, though published, may have not been validated due to the limited time through which they could have been given.

Furthermore, this study does not analyse or discuss a few variables that may alter the COVID-19 pandemic’s influence on neurosurgical training. For example, neurosurgical residency programs worldwide vary in length, structure, workload and caseload, resulting in differences in adaptability among countries, with variable impacts on trainees’ education and programme duration. These variables should be discussed and analysed in future articles, to better understand their impact on training during the pandemic.

## Conclusion

We found all studies to display a disruption in neurosurgical education with emphasis placed upon the fact that trainees have had less clinical and theatre exposure, increased virtual learning and faced considerable challenges with stress, thus resulting in an increased prevalence of mental health issues. Studies further highlighted deployment of trainees to COVID-19 wards being more apparent in LMICs compared to HICs with higher health risks associated with the former, and inequalities in access, affordability and uptake when implementing virtual education platforms in LMICs. Despite this, the shift to online learning has allowed more free revision schedules for trainees, with affordable opportunities such as telesurgery being shown to be useful in all studies. Not only has the pandemic highlighted many issues needing addressing within the current governance of medical education globally, but it has also demonstrated significant companionship between students, residents and educators alike. Encouraging initiatives to further ease demanding physical and mental stress in trainees should be of sheer importance.

## Supplementary Information

Below is the link to the electronic supplementary material.

Supplementary file1 (DOCX 222 kb)

## Data Availability

Systematic review search data is available upon request.
