# Supplementary material for: The impact of the COVID-19 pandemic on global neurosurgical education: a systematic review
Source: Neurosurg Rev. 2021 Oct 8;45(2):1101–10. doi: 10.1007/s10143-021-01664-5 (PMC8497188; doi:10.1007/s10143-021-01664-5)
Supplement: Supplementary file 1 — Supplementary file1 (DOCX 222 kb) [file 10143_2021_1664_MOESM1_ESM.docx]

Supplement 1:

Search strategy:

Search: (COVID-19 OR Sars-CoV-2 OR Coronavirus OR Pandemic) AND (neuro*) AND (Education OR Training OR Training Program OR Teaching)

Filters: Date: 2019 – 2020

Supplement 2:

Table displaying the modifications implemented on Neurosurgical teaching for trainees in the case of each cited studies, with the exception of 3 included studies which targeted medical students (Clifton et al., Jack et al., Thum DiCesare et al.).
Colour code: green signifies that the change has been adopted in the study; red signifies that the change has not been adopted in the study.

| **Study** | **Type of countries (LMIC/HIC)** | **Modifications implemented** | | | | | | | | | | |
| --- | --- | --- | --- | --- | --- | --- | --- | --- | --- | --- | --- | --- |
|  |  | **Online teaching** | **Online conferences/webinars** | **Redistribution/Redeployment to non-neurosurgical units** | **Decreased elective surgical cases** | **Decreased time in the OR/hospital** | **Increased time for research/studying** | **Decreased operative/manual/personal skills** | **Missed electives/fellowships/observerships/rotations** | **Reduced number of residents in the department** | **Exam postponement/cancellation** | **Predicted negative impact on future training** |
| Al-Ahmari et al 2020 | HIC |  |  |  |  |  |  |  |  |  |  |  |
| Alhaj et al 2020 | HIC, LMIC |  |  |  |  |  |  |  |  |  |  |  |
| Aljuboori et al 2020 | HIC |  |  |  |  |  |  |  |  |  |  |  |
| Ashry et al 2020 | LMIC |  |  |  |  |  |  |  |  |  |  |  |
| Burks et al 2020 | HIC |  |  |  |  |  |  |  |  |  |  |  |
| Cheserem et al 2020 | LMIC |  |  |  |  |  |  |  |  |  |  |  |
| Cole T. Lewis et al 2020 | HIC |  |  |  |  |  |  |  |  |  |  |  |
| Dash et al 2020 | LMIC |  |  |  |  |  |  |  |  |  |  |  |
| David T. Fernandes Cabral et al 2020 | HIC |  |  |  |  |  |  |  |  |  |  |  |
| El-Ghandour et al 2020 | HIC, LMIC |  |  |  |  |  |  |  |  |  |  |  |
| Gallardo et al 2020 | LMIC |  |  |  |  |  |  |  |  |  |  |  |
| Khalafallah et al 2020 | HIC |  |  |  |  |  |  |  |  |  |  |  |
| Lazaro et al 2020 | HIC |  |  |  |  |  |  |  |  |  |  |  |
| Meybodi et al 2020 | LMIC |  |  |  |  |  |  |  |  |  |  |  |
| Pelargos et al 2020 | HIC |  |  |  |  |  |  |  |  |  |  |  |
| Rasouli et al 2020 | HIC, LMIC |  |  |  |  |  |  |  |  |  |  |  |
| Saad et al 2020 | HIC |  |  |  |  |  |  |  |  |  |  |  |
| Singh et al 2020 | LMIC |  |  |  |  |  |  |  |  |  |  |  |
| Swiatek et al 2020 | HIC |  |  |  |  |  |  |  |  |  |  |  |
| Wittayanakorn et al 2020 | LMIC |  |  |  |  |  |  |  |  |  |  |  |
| Zoia et al 2020 | HIC |  |  |  |  |  |  |  |  |  |  |  |
